# Tuftsin-Phosphorylcholine Maintains Normal Gut Microbiota in Collagen Induced Arthritic Mice

**DOI:** 10.3389/fmicb.2017.01222

**Published:** 2017-07-10

**Authors:** Hila Ben-Amram, Tomer Bashi, Nir Werbner, Hadar Neuman, Mati Fridkin, Miri Blank, Yehuda Shoenfeld, Omry Koren

**Affiliations:** ^1^Faculty of Medicine, Bar-Ilan UniversitySafed, Israel; ^2^Zabludowicz Center for Autoimmune Diseases, Sheba Medical Center, Sackler Faculty of Medicine, Tel Aviv UniversityTel-Aviv, Israel; ^3^Department of Organic Chemistry, The Weizmann Institute of ScienceRehovot, Israel

**Keywords:** tuftsin-phosphorylcholine, collagen-induced arthritis, rheumatoid arthritis, helminths, microbiome

## Abstract

Rheumatoid arthritis (RA) is characterized by chronic autoinflammation of the joints, with a prevalence of about 1% in Western populations. Evidence in recent years has linked RA to changes in the gut microbiota (dysbiosis). Interestingly, helminths have been shown to have therapeutic activity in RA. Specifically, a glycoprotein containing phosphorylcholine (PC) extracted from helminths was found to have immunomodulatory activity. We have previously developed a novel chimeric compound composed of tuftsin-PC (TPC) that attenuates the joint destruction in mice with collagen-induced arthritis (CIA). Here, we address the interrelationship between TPC immunomodulatory activity and the gut microbiota in CIA mice. Preventive therapy with TPC in mice with arthritis maintained a physiological arthritis score as well as a steady gut microbial environment, similar to that of healthy controls, in contrast to CIA mice with severe disease. The microbial composition differed significantly between healthy and phosphate-buffered saline-treated CIA mice, enabling classifying test samples by machine learning based on levels of a small number of bacterial species. Using these bacterial biomarkers, all TPC-treated CIA mice were classified as healthy. Thus, we describe a clear correlation between TPC treatment, healthy gut microbial communities, and prevention of arthritis. This is the first study to demonstrate the immunomodulatory effect of helminth derivatives in autoimmune diseases and the link to gut microbiota.

## Introduction

The gut microbiota has been extensively studied in recent years, and its impact on lives in both health and disease is only beginning to be understood (Shamriz et al., 2016). Shifts in these gut microbial communities have been linked with a growing list of diseases, including autoimmune diseases (Shamriz et al., 2016). Factors such as diet, age, pregnancy, stress, gender, and even the presence of helminths have all been shown to influence the composition of the microbiome. Interestingly, these factors also influence the risk for autoimmunity (Shamriz et al., 2016).

Rheumatoid arthritis (RA) is a chronic autoinflammation of the joints, affecting about 1% of the Western population (Cross et al., 2014). The disease is typically articular and manifests as stiffness, pain, and swelling of the joints. Recent studies in the emerging field of microbiome research have linked RA to changes in microbiota composition (Shamriz et al., 2016; Neuman and Koren, 2017), suggesting a factor that might be involved in the increased disease prevalence.

The interaction between the microbiome and the immune system is complex and bidirectional, governed by numerous molecular pathways. RA appears to be a good example of these intricate interactions. Metagenomic sequencing demonstrated changes in the compositions of oral and intestinal microbiomes of RA patients compared to controls. This includes overrepresentation of *Prevotella* species (Scher et al., 2013), increased numbers of *Clostridium asparagiforme, Gordonibacter pamelaeae, Eggerthella lenta*, *Bifidobacterium dentium, Lactobacillus* sp. and *Ruminococcus lactaris* (Zhang et al., 2015) and a decrease in *Bacteroides* species (Scher et al., 2013). Interestingly, these changes were partially reversed following treatment with disease-modifying anti-rheumatic drugs (Zhang et al., 2015).

Additionally, numerous studies have investigated the various pathways by which helminths interact with the host immune system. These include promotion of T helper-2 (Th2) inhibition of Th1/Th17 differentiation, amplification of T-regulatory (Treg) and B-regulatory (Breg) cells and type 2-macrophages, skewing of dendritic cells (DCs) toward a tolerogenic phenotype, downregulation of type-2 innate lymphoid cells (ILC2), and modulation of the gut microbiota (Ben-Ami Shor et al., 2013). These immune interactions might explain the successful application of helminths and their ova in the treatment of a large number of autoimmune diseases, including multiple sclerosis, RA, Type 1 diabetes, and inflammatory bowel disease.

Recently, growing evidence has been obtained for the role of helminths in shaping the microbial community in mouse models and human populations, suggesting that helminths may have an impact on the diversity, structure, and function of the gut bacterial community. For example, infection with the mouse parasite *Heligmosomoides polygyrus* leads to higher abundances of *Lactobacillaceae* and *Enterobacteriaceae* in the gut (Gause and Maizels, 2016). Helminths were also found to protect Nod2 Nucleotide-binding oligomerization domain containing 2 (Nod2) knockout mice from colonization by an inflammatory *Bacteroides* species, by inducing type 2 immunity (Ramanan et al., 2016). Interestingly, the relationship between helminths and microbiota composition appears to be reciprocal and multidirectional, since the microbiome can also modulate helminth colonization and survival within the host, and even alter the susceptibility to helminth infection (Reynolds et al., 2015). For instance, it was found that raising levels of *Lactobacillaceae* can increase susceptibility to infection (Ramanan et al., 2016).

Several nematodes, such as the rodent filarial *Acanthocheilonema viteae*, secrete phosphorylcholine (PC)-containing glycoproteins, which have been shown to have anti-inflammatory effects (Harnett et al., 2008). We therefore chose to utilize this component by constructing a chimeric molecule, tuftsin-PC (TPC), composed of PC and an immunomodulatory peptide named tuftsin. Tuftsin is a natural tetrapeptide (Thr-Lys-Pro-Arg), derived from enzymatic cleavage of the IgG-heavy-chain molecule in the spleen (Bashi et al., 2016). Tuftsin has important immunomodulatory effects including an influence on phagocytic cells, antimicrobial, and antiviral properties (Siemion and Kluczyk, 1999). Recently, we showed that TPC treatment delayed glomerulonephritis onset in lupus prone mice (Bashi et al., 2016), prevented colitis in dextran sodium sulfate (DSS) induced colitis (Ben-Ami Shor et al., 2015) and attenuated joint destruction in mice with collagen-induced arthritis (CIA) (Bashi et al., 2016).

Here, we address the effect of TPC on the microbiota and on arthritis severity of treated CIA mice. We show that TPC treatment reduces arthritis in CIA mice, supporting the potent therapeutic effect of this compound. We further show that while the microbial populations of CIA mice treated with phosphate-buffered saline (PBS) alone are dramatically different from those of healthy mice, the CIA mice treated with TPC maintain a steady microbial environment, similar to that of healthy control mice.

## Results

### TPC Treatment Prevents Arthritis in Mice with CIA

**FIGURE 1:**
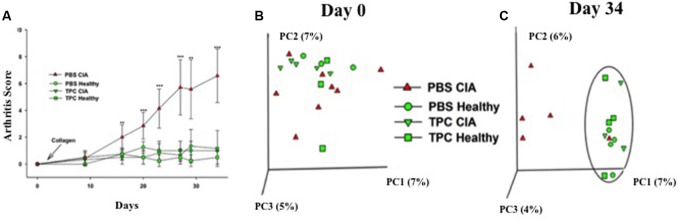
TPC lowers the arthritis score and alters gut microbiota in PBS-CIA mice. **(A)** Arthritis score of healthy (*n* = 4/group) and CIA mice (*n* = 7/group) subcutaneously treated with TPC vs. PBS was recorded at 8 time-points throughout the experiment, starting at day 0 (2 days prior to disease induction) and ending at day 34. Values are presented as the mean ± SD. ^∗∗^*p* < 0.01, ^∗∗∗^*p* < 0.001. **(B,C)** Principal coordinate analysis (PCoA) of fecal microbial communities in CIA and healthy mice treated with TPC or PBS. Community similarity was calculated using the UniFrac distance measure. While no specific clustering by experimental group is evident at day 0 **(B)**, there appear to be 2 clusters at day 34 **(C),** one representing the microbiota from PBS-treated CIA mice, and the other clustering together the TPC-treated CIA mice, TPC-treated healthy mice, and PBS-treated healthy mice (circled), exhibiting similar bacterial populations.

In order to test the effectiveness of the TPC treatment on arthritis, we repeated our previous experiment (Bashi et al., 2016) and compared the arthritis levels of four experimental groups: Collagen-induced arthritis (CIA) mice treated with subcutaneous TPC (TPC-CIA), CIA mice treated with PBS as a control (PBS-CIA), and healthy mice treated with TPC (TPC-healthy) or PBS (PBS-healthy). As detailed in **Figure 1A**, we observed a significantly lower arthritis score in TPC-CIA mice compared to PBS-CIA mice. PBS-CIA mice developed edema and erythema from the ankle to the entire leg, while TPC-CIA mice exhibited milder symptoms. When the CIA mice were sacrificed at day 34, the PBS-treated mice had a mean arthritis score of 6.57 ± 1.98, whereas the TPC-CIA treated mice had a mean arthritis score of 1.16 ± 1.34 (*p* < 0.001). Thus, the TPC treatment in CIA mice reduced arthritis scores to those of the healthy groups of mice not exposed to CIA (0.5 ± 0.5 for PBS-healthy, and 1 ± 0 for TPC-healthy).

### The Gut Microbiota of PBS-CIA Mice Differs from that of Healthy Mice

To determine the effect of TPC on the microbiota of CIA mice, fecal samples were collected every other day, from day 0 until day 34 when the mice were sacrificed. We first compared the baseline compositions of the microbiota at day 0, and verified the similarity between the groups. We then analyzed the composition of the microbiota in the treated and untreated populations. On day 34, we noticed a significant difference between the microbial populations of the PBS-CIA mice versus the TPC-CIA and the TPC-healthy and PBS-healthy groups, which clustered together (**Figures 1B,C**). The difference in the composition of the microbial communities was correlated with disease severity in these groups.

**FIGURE 2:**
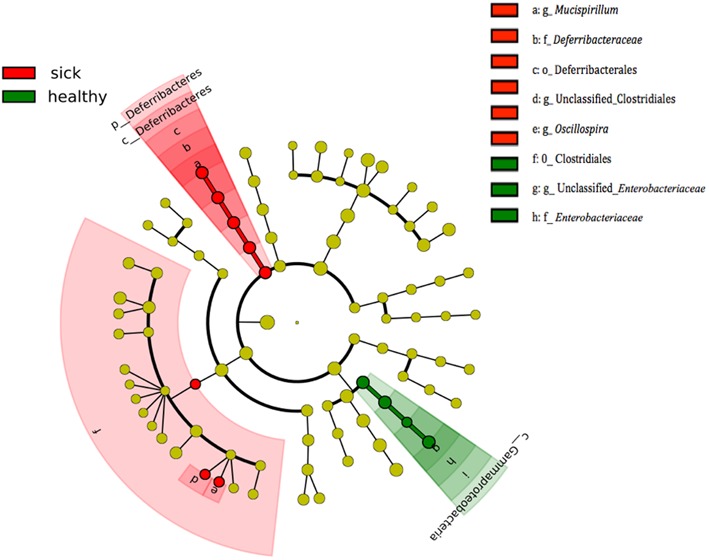
Differentially expressed taxa between healthy and sick (PBS-CIA) mice at day 34. Cladogram indicating the phylogenetic distribution of microbial lineages associated with treatment group. Differences are represented by the color of over-represented bacteria: red indicating the healthy mouse groups (TPC-CIA, PBS-healthy and TPC-healthy mice), green indicating the sick mouse group (PBS-treated CIA mice). Circles represent phylogenetic levels from phylum (outermost circle) to species (innermost circle).

We analyzed the differences between microbiota of the healthy (all three groups) vs. PBS-CIA (sick) animals on day 34 by LefSe (**Figure 2**). At the order level, Enterobacteriales were significantly more (LDA score = 4.8, *p* < 0.05) abundant in the healthy mice while Clostridiales and Deferribacterales were more abundant (LDA score = 3.48, *p* < 0.01) in the PBS-CIA mice (**Figure 2**). At the genus level, *Mucispirillum* was significantly more abundant in the PBS-CIA mice group (LDA score = 3.48, *p* < 0.01, **Figure 2**).

### TPC Treatment Maintains Normal Fecal Microbiota

**FIGURE 3:**
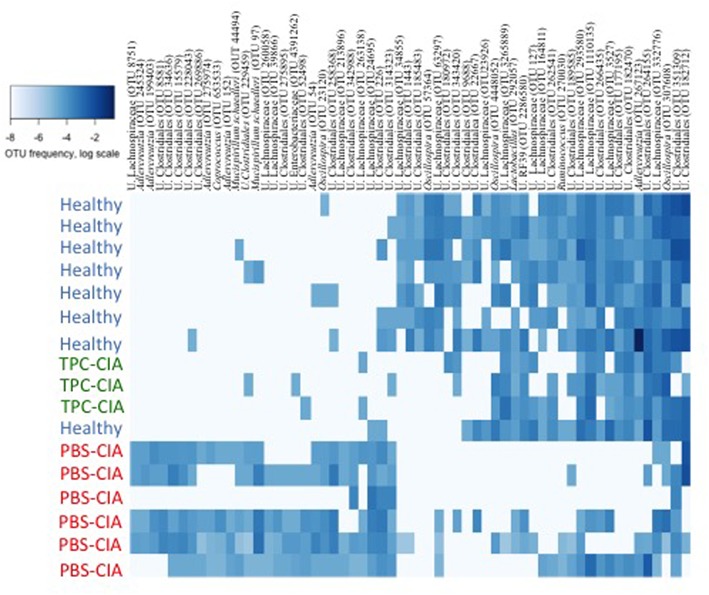
Heatmap of OTU abundances in feces of TPC-CIA vs. PBS-CIA and healthy mice. Heatmap constructed by machine learning at day 34 showing the relative proportions of microbial lineages. Darker shades of blue indicate a higher abundance of an OTU on a logarithmic scale. The machine-learning algorithm was trained on the healthy and PBS-CIA groups, and tested on the TPC-CIA group, with all test samples classified as healthy. U, Unclassified. PBS-CIA mice (red, *n* = 6); TPC-CIA mice (green, *n* = 3); Healthy - TPC or PBS-treated healthy mice (blue, *n* = 8). OTUs are presented by annotation and OTU number.

We further characterized the bacterial profile of the TPC-treated CIA mice on day 34. To this end, we applied a machine-learning algorithm trained on healthy mice (both PBS-healthy and TPC-healthy groups) versus CIA-PBS mice, and then tested the TPC-CIA group. The algorithm classified all TPC-CIA mouse samples to the healthy bacterial signature (**Figure 3**). More specifically, we observed 33 operational taxonomic units (OTUs) that were significantly more abundant in PBS-CIA than in the healthy group, including unclassified Clostridiales, unclassified *Lachnospiraceae*, *Mucispirillum schaedleri*, and *Ruminococcus gnavus.* Additionally, we observed 17 OTUs that were significantly higher in the healthy group, including unclassified Clostridiales, unclassified *Lachnospiraceae*, *Adlercreutzia*, *Coprococcus*, *Roseburia*, and unclassified S24-7. Based on these specific OTUs, the microbiota of TPC-treated CIA mice matched the profile of the healthy mice. Thus, treatment with TPC maintains a healthy microbiota profile, as opposed to the CIA profile seen in the RA mouse control group treated with PBS.

## Discussion

Here, we evaluated the impact of TPC treatment on the microbial communities inhabiting the intestine of CIA mice, and the correlation of the microbiota changes with the clinical outcomes of the treatment. We found a tight correlation between the improved clinical course and the healthy intestinal communities seen following TPC treatment. In contrast, a unique microbiota composition, distinct from the healthy one, was correlated with the CIA disease state.

Clinically, we found that mice with CIA that were treated with TPC displayed low arthritis scores as did both healthy controls (treated or not with TPC), while the mice with CIA that were treated with PBS alone displayed significantly higher scores indicating a disease state. These results are consistent with our previous findings demonstrating the potential of TPC to prevent the development of arthritis in CIA mice, manifested by attenuation of joint destruction, and a reduced overall inflammatory state (Bashi et al., 2016).

Analysis of the gut microbiota composition revealed alterations in CIA *vs*. healthy mice. However, CIA mice treated with TPC maintained a “healthy” microbiota, similar to that of mice in which RA was not induced. Our results revealed a higher abundance of *Mucispirillum, Oscillospira*, and unclassified Clostridiales genera in fecal samples of PBS-CIA mice at the expense of *Enterobacteriaceae*. More specifically, 33 OTUs were significantly more abundant in PBS-CIA than in the healthy group, including unclassified Clostridiales, unclassified *Lachnospiraceae*, *M. schaedleri* and *Ruminococcus gnavus*, while 17 OTUs were significantly higher in the healthy group, including unclassified Clostridiales, unclassified *Lachnospiraceae*, *Adlercreutzia*, *Coprococcus*, *Roseburia*, and unclassified S24-7. These findings are consistent with other autoimmune models in mice also demonstrating significant changes in microbiota in correlation with disease progression. For example, in a murine model of infectious colitis, early disruption of the colonic surface mucus layer, which appears prior to the onset of symptomatic colitis, was associated with increased *Mucispirillum* abundance (Belzer et al., 2014). Moreover, DSS, which induces colitis in mice, was shown to increase the relative abundance of the bacterial families *Ruminococcaceae*, *Bacteroidaceae*, *Enterobacteriaceae*, *Deferribacteraceae*, and *Verrucomicrobiaceae* as well as the putative mucin degraders *Akkermansia* and *Mucispirillum* (Berry et al., 2015). Specifically, *M. schaedleri* is associated with the inflammatory environment, possibly due to its ability to reduce nitrate and express systems for scavenging oxygen and reactive oxygen species (Loy et al., 2017). However, it is not yet clear whether *M. schaedleri* plays an active role affecting the inflammatory state, and if so, whether the presence of this species enhances or alleviates inflammation. It has been suggested that *M. schaedleri* may play an anti-inflammatory role during inflammation by modifying gene expression in host mucosal tissue (Loy et al., 2017).

An interesting observation is the significant enrichment of short chain fatty acid (SCFA) producers in healthy mice compared to diseased CIA mice. For example, members of the *Lachnospiraceae* family, which are known propionate producers are increased in the healthy mice. *Coprococcus* and *Roseburia*, both of which are also more abundant in healthy mice, are examples of a small group of bacteria that are known to produce both Butyrate and propionate, as most SCFA producers produce only one SCFA (Rios-Covian et al., 2016). As SCFAs have anti-inflammatory properties, the disappearance of these SCFA producing bacteria from the microbiota of diseased mice may be linked to the inflammatory response seen in the disease state. More studies are needed to determine whether these microbial changes are a cause or consequence of the CIA.

The distinct microbiota composition in the untreated CIA mice versus TPC-treated CIA mice and healthy controls, suggest dysbiosis as an additional symptom in CIA. Moreover, since machine learning clearly distinguishes between TPC-treated and untreated CIA mice, microbial composition may serve as a biomarker for treatment success, correlated with improved overall symptoms.

The similarities in microbial profiles observed between the healthy-PBS and healthy-TPC groups demonstrate that TPC does not have a direct effect on the microbiota composition. Therefore, our results suggest that TPC initially affects the immune system, mitigating autoimmunity, and that subsequently, once the disease is controlled, the microbiota returns to its normal healthy state. TPC is thought to accelerate expression of anti-inflammatory cytokines and expansion of Treg cells. The TPC itself is a bi-specific molecule, composed of PC, the immunomodulatory molecule presented by secretory glycoproteins of the helminths, which suppresses the TLR4 related cascade, and tuftsin, a self peptide produced by the spleen that affects the immune cell function via neuropilin and Fcγ receptors (unpublished data). As we have previously shown the effectiveness of TPC in prevention of experimental models of lupus and DSS-induced colitis (Bashi et al., 2015; Ben-Ami Shor et al., 2015), we believe that the mechanism of TPC, involving immunomodulation and microbial changes, is likely to be more general and relevant to other medical conditions in addition to RA.

Overall, in this report we describe a clear correlation between TPC treatment, healthy gut microbial communities, and prevention of arthritis. This is the first study to demonstrate the immunomodulatory effect of helminth derivatives in autoimmune diseases and the link to gut microbiota. While future research with a group sizes is needed to fully characterize the roles of the specific species and higher taxonomic levels altered in the disease state and how TPC restores the gut microbiota, this study provides an essential and initial step in this process.

## Materials and Methods

### Tuftsin-Phosphorylcholine Synthesis (TPC)

TPC was synthesized as previously described (Bashi et al., 2015, 2016). Basically, tuftsin was extended at the C-terminal, i.e., Thr-Lys-Pro-Arg-Gly-Tyr, and synthesized manually following solid-phase peptide technology (GLS peptide synthesis; GLBiochem, Shanghai, China). The peptide was then coupled to a diazotized 4-aminophenyphosphorylchloride to form an azo bond between the tuftsin and the PC (Sigma–Aldrich, St. Louis, MO, United States) (Koch et al., 1973; Fridkin and Najjar, 1989). The conjugate was then characterized by MS and amino acid analysis as well as by HPLC. TPC was suspended in commercial PBS (Biological Industries, Israel Beit-Haemek Ltd., Kibbutz Beit-Haemek, Israel).

### Mice and Experimental Design

Experimental arthritis was induced in DBA/1 male mice at the age of 6–7 weeks (Harlan laboratories, Kreuzelweg, Netherlands). The mice were maintained in a conventional animal housing facility at Sheba Medical Center in individually ventilated cages. All experiments were approved by the Ethics Committee of the Israeli Ministry of Health no.696/11. Disease was induced by intradermal injection of 100 μg bovine type II collagen (Chondrex) in 1:1 emulsion with *Mycobacterium tuberculosis* H37RA in Freund’s incomplete adjuvant (Difco Laboratories) into the base of the tail. A boost injection of bovine type II collagen in PBS at the base of the tail was given after 2 weeks. Mice were divided into two groups –TPC treated, and PBS treated controls. In each treatment group, seven mice were immunized with collagen type II, of which all developed CIA, and four mice were not immunized and considered healthy.

TPC or PBS (vehicle) was first administered at day 0, 2 days prior to disease induction. TPC was injected subcutaneously (s.c) (5 μg/0.1 ml per mouse) twice a week. The mice were sacrificed after 34 days.

### Assessment of Arthritis

Mice were monitored for signs of arthritis three times a week by two observers blinded to the treatment group; severity scores were defined as follows: 0 = normal, 1 = slight erythema, 2 = slight erythema plus swelling, 3 = moderate edema and erythema 4 = edema and erythema from the ankle to the entire leg. The total arthritis score was the sum of the scores of each of the four limbs.

### Bacterial DNA Extraction, Amplification and Sequencing

DNA from stool samples was extracted using the Power Soil DNA Isolation Kit for 96 well plates (MoBio, United States) according to the manufacturer’s instructions. The V4 region of the bacterial 16S rRNA gene was amplified with the 515F and 806R barcoded primers, and the products purified, quantified, and pooled together at equal concentrations (50 ng/μl), as previously described (Caporaso et al., 2012). Purified products were sequenced using the Illumina MiSeq platform (Genomic Center, Faculty of Medicine, BIU, Israel).

### Microbiome Analysis

Data analysis was performed using QIIME version 1.8.0 (Caporaso et al., 2010), as described previously (Reed et al., 2015). Paired–end sequences were joined using fastq-join, demultiplexed, and quality filtered with an average quality threshold of 25. Chimeric sequences were removed, and reads were clustered into OTUs using the open reference UCLUST method (Edgar, 2010) against the GreenGenes 08/13 database (DeSantis et al., 2006), with a cutoff of 97% sequence identity. Core OTUs were calculated by filtering for OTUs present in at least 50% of subjects in the same treatment group. Analyses were performed on a rarefied table of 11,000 sequences per sample. In addition, Alpha diversity was calculated using the Chao1 measure, and beta diversity was analyzed using UniFrac (Lozupone and Knight, 2005). Machine learning was done using the shrunken centroids method on log frequencies of OTUs using the PAMR package in R (Tibshirani et al., 2002). The algorithm was trained on day 34 samples from healthy and CIA groups, and used for prediction on all other samples. Differential OTUs between groups and cladograms were generated using LEfSe (Segata et al., 2011).

## Data Availability

The 16S rRNA gene sequence data have been deposited in the European Bioinformatics Institute (EBI) database with accession code ERP023025.

## Author Contributions

HB-A, TB, NW, HN, MF, and MB performed the experiments and analyzed the data. HN, MB, YS, and OK wrote the manuscript.

## Conflict of Interest Statement

YS and MB are shareholders in TPCera. The other authors declare that the research was conducted in the absence of any commercial or financial relationships that could be construed as a potential conflict of interest.
